# An Update on Silent Corticotroph Adenomas: Diagnosis, Mechanisms, Clinical Features, and Management

**DOI:** 10.3390/cancers13236134

**Published:** 2021-12-06

**Authors:** Shenzhong Jiang, Xiaokun Chen, Yinzi Wu, Renzhi Wang, Xinjie Bao

**Affiliations:** Pituitary Center, Department of Neurosurgery, Peking Union Medical College Hospital, Chinese Academy of Medical Sciences, Peking Union Medical College, Beijing 100730, China; pumc_shenzhongj@student.pumc.edu.cn (S.J.); xiaokunchen123@outlook.com (X.C.); pumc_wuyinzi@student.pumc.edu.cn (Y.W.)

**Keywords:** silent corticotroph adenoma, non-functioning pituitary adenoma, transcription factors, mechanisms, clinical features

## Abstract

**Simple Summary:**

The 2017 World Health Organization classification of endocrine tumors defines pituitary adenomas based on their cell lineages. T-PIT can serve as a complimentary tool for further identification of silent corticotroph adenomas (SCAs). Unlike functioning corticotroph adenomas in patients with Cushing’s disease, SCAs present no clinical and biochemical features of Cushing’s syndrome. SCAs have been shown to exhibit a more aggressive course characterized by a higher probability of recurrence and resistance to conventional treatment due to their intrinsic histological features. The aim of our review is to offer an update on the diagnosis, mechanisms, clinical features and management of SCAs. Studies of the molecular mechanisms of SCA pathogenesis will provide new directions for the diagnosis and management of SCAs.

**Abstract:**

With the introduction of 2017 World Health Organization (WHO) classification of endocrine tumors, T-PIT can serve as a complementary tool for identification of silent corticotroph adenomas (SCAs) in some cases if the tumor is not classifiable by pituitary hormone expression in pathological tissue samples. An increase of the proportion of SCAs among the non-functioning pituitary adenomas (NFPAs) has been witnessed under the new rule with the detection of T-PIT-positive ACTH-negative SCAs. Studies of molecular mechanisms related to SCA pathogenesis will provide new directions for the diagnosis and management of SCAs. A precise pathological diagnosis can help clinicians better identify SCAs. Understanding clinical features in the context of the pathophysiology of SCAs is critical for optimal management. It could provide information on appropriate follow-up time and aid in early recognition and treatment of potentially aggressive forms. Management approaches include surgical, radiation, and/or medical therapies.

## 1. Introduction

Silent corticotroph adenomas (SCAs) are a subtype of non-functioning pituitary adenomas (NFPAs). Unlike functioning corticotroph adenomas in patients with Cushing’s disease (CD), SCAs present no clinical and biochemical features of Cushing’s syndrome. 

Previous studies have shown SCAs account for 4.8–6.8% of all resected pituitary adenomas (PAs), 5–19% of all NFPAs, and up to 20% of all corticotroph adenomas [1,2,3,4,5,6].

The diagnosis of SCAs is made in a retrospective fashion with histopathological staining since clinical and endocrinological characteristics cannot distinguish them from other NFPAs. They are traditionally diagnosed by observation of positive immunoreactivity for adrenocorticotropic hormone (ACTH). 

The 2017 World Health Organization (WHO) classification of endocrine tumors recently defined PAs based on their cell lineages [7]. It is generally acknowledged that cell lineage-specific pituitary transcription factors play a crucial role in the development of pituitary cells and corresponding PAs: T-box family member TBX19 (T-PIT) for corticotroph lineage; pituitary-specific POU-class homeodomain transcription factor (PIT-1) for somatotroph, lactotroph, and thyrotroph lineages; and steroidogenic factor 1 (SF1) for gonadotroph lineages [8]. Therefore, in light of the new WHO classification, the diagnosis of SCAs remains based on immunohistochemistry (IHC) for ACTH. However, if an adenoma, particularly for NFPA, is still not classifiable by pituitary hormone expression according to a cell lineage, assessment of transcription factors may serve as a second-step complimentary tool for further classification, e.g., T-PIT in the case of SCAs. 

SCAs are reportedly highly proliferative and invasive [3,9,10,11,12]. They have been shown to exhibit a more aggressive course characterized by a higher probability of recurrence and resistance to conventional treatment and are therefore classified as “high-risk” tumors according to the 2017 WHO classification system [6,7,13].

This review provides an update on the SCA diagnostic approach; novel molecular mechanisms related to SCA pathogenesis, which are implicated in ACTH silence, growth, invasion, and aggressive behavior; and a summary of the clinical characteristics of SCAs and their management. 

## 2. Identification of SCAs

With the implementation and wider use of T-PIT IHC, the proportion of SCAs among all NFPAs is expected to increase if T-PIT-positive ACTH-negative SCAs can be identified. Nishioka et al. studied 119 previously diagnosed hormone-negative tumors by evaluating the expression of transcription factors. The authors were able to reclassify 95% of them as silent pituitary tumors, of which 26.9% (32/119) were reclassified as SCAs as they displayed differentiation towards the corticotroph lineage with T-PIT immunopositivity. The IHC studies of transcription factors have drastically reduced the percentage of null-cell tumors (from 23% to 1%) and further increased the percentage of SCAs (from 10% to 16%) from the surgically resected NFPAs [14]. It is noteworthy that ACTH remains the first line for detection of SCAs. Nevertheless, for laboratories using less sensitive ACTH antibodies, or using automatic platforms without antigen retrieval protocols IHC, T-PIT may serve as a second-step complimentary tool for identification of SCAs. 

Some studies on SCAs have been conducted following the 2017 WHO classification guidelines [9,15,16,17,18,19]. All have seen an increase of the proportion of SCAs among the NFPAs with the detection of T-PIT-positive ACTH-negative SCAs as T-PIT immunostaining reduces the number of false-negatives and false-positives. However, the implementation of T-PIT IHC in clinical practice is not without its pitfalls. Though anti-T-PIT antibodies (HPA072686) that are both sensitive and specific have been developed [20], it is not available in all institutions, and in addition, the unavailability of well-trained pathologists for IHC techniques restricts the interpretation of transcription factors to a few laboratories and platforms [21]. Even though some institutions have performed the T-PIT IHC, variability in the results among different departments has been observed and their reliability may be questioned [22]. Furthermore, the sensitivity of IHC in identifying the variant subtypes of tumors may be decreased as the protein expression of a specific gene is sometimes aborted. 

To overcome the limitations of IHC and to complement it at the same time, Torregrosa-Quesada et al. [23] from Alicante General University Hospital-Institute for Health and Biomedical Research (ISABIAL), Spain, targeted the quantification of transcription factor gene expression by RT-qPCR to identify the cellular origin of pituitary tumors. In all tumor samples, they analyzed the gene expression of the main transcription factors associated with three cell lineages. Based on the clinically functioning pituitary adenomas, the authors established the interquartile range (p25–p75) of relative expression for all genes studied in each subtype. Among silent adenomas, when there is only the dominant gene of the corticotroph cell lineage, i.e., only a fold change (by RT-qPCR) of *T-PIT* above p25, the tumor is diagnosed as SCAs, the silent counterparts of functional corticotroph adenomas. They also studied the protein expression of transcription factors in a subset of 56 tumors to calculate the concordance between gene and protein expression. The concordance (Cohen’s kappa coefficient is 0.913) proved high between T-PIT immunopositivity and *T-PIT* expression. 

We also validated the transcription factors at the RNA level in a subset of 61 NFPAs (25 of them were SCAs) after they were reclassified according to transcription factor IHC. We demonstrated good concordance between the molecular and IHC identification of the NFPAs subtypes [18]. According to these results, the molecular characterization of *T-PIT* complements the IHC results, and whenever IHC study of T-PIT is not available, RT-qPCR of *T-PIT* could help pathologists, neurosurgeons, and endocrinologists for better identification of SCAs. Further classification of PAs may be dependent on RT-qPCR of transcription factors that is precise, with little bias and unified standardization among different pathology departments. 

## 3. Pathogenesis

### 3.1. Why Silent?

There are several hypotheses on the silencing mechanism of SCAs. Kovacs et al. [24] observed that SCA cells have a high number of lysosomes in the cytoplasm and show fusion of these lysosomes with secretory granules as evidenced by electron microscopy, leading to the hypothesis that ACTH is destroyed before it can be released. The possibility that a defective *POMC* gene was responsible for the silent nature of SCAs was denied by ribonuclease mapping analysis as no mutation was detected of *POMC* [25]. There is also speculation that SCA cells may not derive from anterior pituitary lobe ACTH-expressing cells, which give rise to CD cells, and rather they may arise from the pars intermedia POMC-expressing cells. Therefore, SCAs may have distinct characteristics from CD adenomas [26]. We will discuss in the next section the origin of SCAs. Others suggest that SCAs secrete predominantly biologically inactive high-molecular-weight ATCH to compete with normal ACTH (1–39) at the ACTH receptor, thus silencing corticotroph adenomas [27]. 

Recent advances have focused on the transcription, post-transcriptional regulation of *POMC*, and post-translational regulation of ACTH to explain the silencing mechanism of SCAs. 

Araki et al. [28] observed that an additional regulatory region near the *POMC* gene, functioning as a second promoter of *POMC*, is highly methylated in SCAs. In contrast, it is highly demethylated in pituitary ACTH-secreting tumors, suggesting that methylation of the second promoter might play a role in transcription of the *POMC* gene, possibly silencing *POMC* in SCAs. 

Given the fact that SCAs and CDs in most studies expressed similar *POMC* [11,29,30], a most likely and feasible hypothesis has been established that the function or expression of prohormone convertase (PC) 1/3, a POMC-processing enzyme, may be disturbed in SCAs. PC1/3 has been extensively studied in the literature [11,29,30,31,32,33,34]. PC1/3, encoded by the *PCSK1* gene, is involved in the processing of *POMC* into mature and biologically active ACTH [34]. Concerning *POMC* post-transcriptional regulation, Tateno et al. [31] and Jahangiri et al. [11] observed that the expression of PC1/3 was present at 15-fold and 30-fold higher levels in CDs than SCAs, respectively, indicating defects in the conversion of *POMC* to ACTH in SCAs. ISABIAL’s lab [29] also observed lower *PCSK1* and PC1/3 expression in SCAs compared with CDs, reinforcing the hypothesis that defective PC1/3 is implicated in the impaired *POMC* post-transcriptional regulation. 

MicroRNAs(miRNAs) have also been proposed to play a potential role in the silencing mechanism of SCAs. miRNome analysis revealed differences between SCAs and CDs as they were arranged in two clusters with a different miRNAs signature, suggesting a potential part played by the miRNAs in the pathophysiology of different corticotroph adenomas [35]. Comparing 23 SCAs and 24 CDs, García-Martínez [1] observed significantly higher levels of miR-200a and miR-103 in SCAs than in CDs (indeed, both miRNAs appeared normo-regulated in SCAs while downregulated in CDs), and suggested these miRNAs may serve as potential diagnostic markers to distinguish SCAs from CDs. In SCAs, miR-200a and miR-103 could participate in the regulation of *POMC* expression. *POMC* transcription is associated with the *PKA-MAP3K8-MEK-ERK1/2-NGFIB* pathway [36], while miR-200a and miR-103 negatively regulated this pathway by inhibiting the *MAP3K8* expression. Additionally, miR-103 correlated negatively with the expression of *MEK.* Therefore, miR-200a and miR-103 are involved in the post-transcriptional regulation of *POMC* expression in SCAs by inhibiting its upstream pathway [1]. Moreover, the authors demonstrated other miRNAs, such as miR-375, miR-488, and miR-383, could participate in the regulation of *POMC* transcription, probably through their association with different pathways and relevant transcription factors. 

Post-translational regulation of ACTH has also been studied [11,29,31,37]. The proconvertase PC2 is proposed to contribute to the silencing mechanism of SCAs. Encoded by *PCSK2*, PC2 is an endoproteolytic enzyme responsible for the processing of biologically active ACTH into α-melanocyte-stimulating hormone and corticotropin-like intermediate lobe peptide in the intermediate lobe [38]. Most studies reported a decreased degradation of ACTH in corticotroph adenomas (SCAs and CDs) compared with other subtypes of NFPAs because PC2 mRNA levels in SCAs and CDs were less than those in other subtypes of NFPAs, without differences between SCAs and CDs [11,31]. Further study by García-Martínez et al., however, found a stronger correlation between *PCSK2* and PC2 expression in SCAs than in CDs. Moreover, the authors found that SCAs exhibited higher *PCSK2* expression than CDs (though not statistically significant). These results are consistent with a more efficient and accelerated degradation of ACTH in SCAs than in CDs, thereby partly accounting for silent manifestations of Cushing’s syndrome in SCAs [29]. In addition, more peptidylglycine α-amidating monooxygenase (PAM) and carboxypeptidase E (CPE) expression in SCAs than in CDs, both involved in the post-translational regulation of ACTH, might lead to the inability to secrete ACTH in these SCAs. 

Another interesting phenomenon is that SCAs can transform into active CDs after years of silence. The phenotypic transition has been related to three mechanisms: first, an increase in *PCSK1* and PC1/3 expression has been reported in three cases of SCAs that evolved during follow-up to functioning ones, thus underscoring the role of PC1/3 as one of the most feasible mechanism for silencing corticotroph adenomas [39]. Second, though decreased in SCAs, the accumulative effect of PC1/3 could still process POMC into biologically active ACTH if time allows. Little by little, ACTH could gradually and eventually rise to a higher level to cause clinical manifestations of Cushing’s syndrome. Indeed, the transformation took a median of 30 months, with the longest interval being 10 years summarized by Zheng et al. [40]. Additionally, a most recent mechanism proposed by Araki et al. [28] suggested that the sequential hypomethylation of the second promoter might activate *POMC* and promote ACTH secretion in the phenotypic transition. 

### 3.2. SCAs Could Represent Silent Cortico-Gonadotroph Adenomas?

Cooper et al. [41] proposed a pathologic and clinically distinct typification of SCAs as silent cortico-gonadotroph adenomas as they exhibit features consistent with both the corticotroph and the gonadotroph cell lines. 

In this study, SCAs have been shown to be immunopositive for corticotroph markers, ACTH, NeuroD1, as well the as gonadotroph markers, SF-1 and DAX-1. Some cells co-expressed ACTH with either SF-1 or LH. In contrast, their secreting counterparts are immunonegative for DAX-1. It is noteworthy that only 44% of SCAs are immunoreactive for T-PIT, which could be attributed to T-PIT antibodies that were not readily available and reliable when the research was conducted. Under electron microscopy, SCAs demonstrated ultrastructural features consistent with both corticotroph and gonadotroph cells. It is thus hypothesized that SCAs derive from an intermediary cell that shares both gonadotroph and corticotroph characteristics. 

Intriguingly, further investigation reported that 20.45% (9/44) of the corticotroph adenomas expressed *GATA2*, a gene of the transcription factor involved in the development of gonadotrophs [23], also considered a marker of gonadotroph adenomas [42]. Eight of the nine cases that expressed *GATA2* are SCAs. The expression of *GATA2* in SCAs suggests that this subtype could have a component of gonadotrophic features [23]. 

Coincidentally, by using a canonical transcriptome signature for each pituitary cell type, Neou et al. observed that 88% (7/8) of SCAs displayed both corticotroph and gonadotroph signatures [35]. It was later confirmed by co-expression of GATA3 (a genomic paralogy of GATA2) to be characteristic of pituitary gonadotroph adenomas [43], ACTH, and T-PIT IHC in these tumors. The result highlights that SCAs manifest both corticotroph and gonadotroph cytologic features, supporting the arguments by some authors [23,41]. Further research by Ricklefs et al. [44] analyzing genome-wide DNA methylation profiles also demonstrated that SCAs show a spatial relationship with gonadotroph adenomas. These findings suggest that SCAs might arise from a specific cell lineage distinct from other corticotroph adenomas and that they could represent a new pituitary tumor subtype named cortico-gonadotroph adenomas, but it still requires further investigations in a larger cohort of SCAs. 

### 3.3. Progression and Growth 

SCAs are among the five types of “high-risk” tumors proposed by the 2017 WHO classification system that demonstrate aggressive behavior. The concept of aggressiveness in SCAs is a composite of high invasion, proliferation, progression, and frequent recurrences. 

Over the past years, advanced techniques have allowed the exploration of either mono-omic or multi-omics, such as genomic, transcriptomic, epigenomic, metabolomic, proteomic, and lipidomic, to study the characteristics of NFPAs [45,46,47,48,49,50,51]. Though attempts to explore the underlying mechanism of invasiveness and aggressiveness of SCAs are still limited and inconclusive, we have already witnessed some advances, especially those using various -omics approaches to provide novel insights into the invasive and aggressive behavior of SCAs. 

Various factors have been implicated in the development of SCA progression and growth including cyclin-dependent kinases inhibitor 2A, galectin-3, kallikrein 10, osteopontin, and O6-methylguanine-DNA methyltransferase. They have been reviewed by Ben-Shlomo et al. [4], but they are merely associative and remain to be further elucidated. Of note, as is pointed out in the 2017 WHO edition [7], galectin-3 has been reported to be weak or absent in SCAs compared with CDs and thus can be used as an important marker to distinguish between the two forms [52]. However, the direct effect of the absence of galectin-3 on SCAs’ behavior remains further elucidated. Since NFPAs are best studied in the emerging multi-omics studies, here, we summarize insights from the recent developments in SCA progression and growth. 

Recently, Wang et al. investigated the invasiveness-associated lipid alterations in SCAs through ultra-performance liquid chromatography-mass spectrometry (UPLC-MS)-based lipidomic analysis [16]. Twenty-eight differential lipids were identified in 54 SCAs (34 invasive/20 noninvasive) and found to be associated with active proliferation of SCAs. Transcriptome analysis was also conducted in another cohort of 42 NFPAs (23 invasive/19 noninvasive). Then, a multiomic functionally connected network was established with two lipids, 17 differentially expressed genes, and four molecular pathways to explore their potential molecular mechanism in the invasiveness of SCAs. These lipids and genes were found enriched in the energy metabolism-related pathway, leading to the invasiveness of SCAs since rapid proliferation and invasion of tumors require the metabolism of a large amount of lipids. Finally, by using multiple machine learning methods, the authors identified the four most critical lipids contributing to the invasive phenotype of SCAs, including three membrane-constituting lipids and one signal-transducing lipid, and thereby constructed a four-lipid risk model, which demonstrated excellent predictive ability to discriminate the invasive SCAs from non-invasive ones. 

Neou et al. [35] generated a pangenomic classification of PAs. In their comprehensive research, transcriptomic analysis revealed low epithelial-mesenchymal transition (EMT) in SCAs along with their functioning *USP8*-mutated counterparts, while increased EMT was seen in their functioning *USP8* wild-type counterparts showing increased invasiveness. As EMT is associated pathologically with tumor invasion and progression, the result might suggest that MET is not the main process that drives for the invasiveness of SCAs, and factors or processes other than MET may play a more crucial role in the proliferation and invasiveness in SCAs. 

Epigenetic mechanisms, such as DNA methylation, have been shown to well distinguish between SCAs and CDs through DNA methylomic analysis [35], which has also been suggested by Ricklefs et al. [44]. DNA methylation of specific genes is related to the growth and invasiveness of PAs [53,54,55]. However, further research is needed to elucidate which key genes are involved in invasiveness-related aberrant epigenetic deregulation that lead to the invasiveness and aggressiveness of SCAs. 

By high-throughput sequencing, weighted gene co-expression network analysis, and functional annotation, we observed gene signatures correlated with the invasiveness of SCAs [18]. Twenty differentially expressed genes were identified enriched in the “Pathway in cancers” and “MAPK pathway” and contributed to alterations of related factors. For instance, several receptor tyrosine kinases (EGFR and NTRK1) and their downstream signal transducers (RRAS and JAK1) showed enhanced expression in invasive SCAs. These components jointly regulate the tumorigenesis of SCAs. We next analyzed three invasion-related molecular markers (INSM1, HSPA2, and CDK6) in three subtypes of NFPAs (SCAs, SGAs, and null-cell adenomas) and found that INSM1 and HSPA2 expressions were higher in SCAs than other subtypes and that CDK6 showed a tendency of positive correlation with invasive SCAs. These results further supported the strong invasiveness of SCAs [18]. 

Uraki et al. studied the mismatch repair genes *mutS homologs 6/2* (*MSH6/2*) and *programmed cell death 1 ligand 1* (*PD-L1*), which are involved in tumor growth and tumor immunity, respectively, in a cohort of 73 NFPAs, 23 of them SCAs [56]. Results showed that reduced expressions of *MSH6/2* and *PD-L1* mRNA in SCAs partially account for the molecular mechanism causing proliferative and invasive characteristics as the reduction of *MSH6/2* could decrease the rate of apoptosis and promote cell-cycle progression, and the reduction of *PD-L1* could provide a relatively functional tumor immunity. However, their detailed mechanism warrants further in-depth studies. 

In corticotroph adenomas, pituitary-specific hormone gene expression of arginine vasopressin receptor 1B (*AVPR1B*) is observed to express higher in SCAs than in CDs. Since arginine vasopressin is capable of activating pathways associated with cell proliferation, higher *AVPR1B* mRNA expression could be related to the growth and aggressiveness of SCAs [29]. However, another study by ISABIAL’s lab found comparable *AVPR1B* mRNA between SCAs and CDs, inconsistent with their previous finding [57]. 

## 4. Clinical Features

### 4.1. Clinical Manifestations

SCAs commonly manifest as NFPAs, presenting with symptoms of mass effects. Patients may present headache, visual symptoms, and hypopituitarism, without the clinical features of Cushing’s syndrome [58]. However, the clinical course of SCAs is different from its name, ‘silent’; rather, they show aggressiveness. Some studies show that SCAs may have a female predominance [10,59,60], while others do not [3,11,61]. Regarding the age of onset, some studies have observed SCAs may present at younger ages than other subtypes of NFPAs [10,12,17,60].

### 4.2. Preoperative Diagnosis

It is difficult to distinguish SCAs from other NFPAs preoperatively. Researchers have attempted to find clinical evidence to detect SCAs before surgery [15,62]. Kim et al. identified that female, tumor hemorrhage, cavernous sinus invasion, and weaken ACTH response in the combined pituitary function test (CPFT) may be associated with SCAs [62]. Meanwhile, Zhang et al. summarized that female, cystic degeneration, and high blood ACTH levels (for ACTH positive SCAs) may serve as reliable predictors for SCAs [15]. Recent studies suggested that lipidomic signature [16] and serum-exosomal INSM1 mRNA [18] may serve as promising diagnostic techniques for the invasive SCA.

### 4.3. Radiologic Characteristics

SCAs usually present as macroadenomas, typical of NFPAs characteristics [4,58]. However, it is not uncommon to find marked cavernous sinus invasion in SCAs than in other NFPAs [11,14,17,60,63], while this finding was not demonstrated in other studies [12,64]. Jahangiri et al. argued that differences may come from relying exclusively on the radiologist’s diagnosis of the preoperative MRI [11]. They addressed that it was indispensable to incorporate a surgeon’s impression on the cavernous sinus invasion. Otherwise, wrong conclusions may be drawn [11]. In addition, Kim et al. revealed that surgical findings should play a decision role in the diagnosis of cavernous sinus invasion when invasiveness could not be confirmed before surgery [65]. Some have observed that the occurrence of multiple microcysts [17,63,64,66] and intertumoral hemorrhage [63,64,66] are more prevalent in SCAs than those in NFPAs, while others not [11,67]. In the group of SCAs and SGAs, the sensitivity and specificity of multiple microcyst for predicting SCAs are 76% and 95%, respectively [68]. However, this study only assessed between SCAs and SGAs, without other types of PAs. Kasuki et al.’s research made up for this shortcoming [69]. They analyzed the frequency of these special microcysts in all subtypes of pituitary adenomas. They concluded that microcystic patterns are more common in SCAs than in other PAs. The sensitivity, specificity, and accuracy to define an SCA are 58%, 93%, and 90%, respectively [69]. The high specificity of multiple microcysts to predict an SCA has also been confirmed by other reports [17].

## 5. Postoperative Course

### 5.1. Postoperative Hypopituitarism

A retrospective series concluded that more than half of the patients newly developed postoperative adrenal insufficiency [3,41]. Ben-Shlomo et al. put forward a hypothesis in their article that development of postoperative hypopituitarism may result from the suppression of normal ACTH [4]. They proved that SCAs may release some tumoral ACTH locally, leading to the insufficiency of ACTH after the resection of tumor [4]. 

### 5.2. Recurrence

A number of studies have investigated whether SCAs have a more aggressive behavior (Table 1). Some studies have demonstrated that SCAs may have higher rates of recurrence than other NFPAs [3,11,17,41,63], while others described there were no significant differences between SCAs and other NFPAs in the time or rates of recurrence [10,12,61,62,64,66,70,71]. These differences may result from the time of follow-up, sample size, and the use of adjuvant radiotherapy [4]. A recent meta-analysis, which included 244 SCAs and 1622 other NFPAs, did not verify that SCAs have higher recurrence rates than NFPAs [70]. Although the recurrence or progression rates between SCAs and other NFPAs may have no statistical significance, SCAs still present a more aggressive clinical course than other NFPAs judging from the mean time to disease progression [10]. Drummond et al. summarized that SCAs may present aggressive behavior and higher recurrences rates than other NFPAs after a follow-up time of more than 3 years [58]. Bradley et al. noted that SCAs may show a more aggressive course when they do regrow [71]. Cho et al. reviewed the medical records of 162 cases diagnosed with NFPAs (28 SCAs and 134 ACTH-immunonegative NFPAs) [63]. They found that the overall recurrence rate was not significant between SCAs and non-SCAs at the beginning, even without the interference of adjuvant radiotherapy. As time goes by, the difference became apparent. After a follow-up time of more than 5 years, the recurrence rates of SCA and non-SCA were 57.1% and 13.9% (*p* = 0.04), respectively. Additionally, their research also suggested multiple recurrences and aggressive tumor behavior may be associated with younger age [63]. They highlighted the importance of careful long-term monitoring for patients who are diagnosed at a young age [63]. However, other reports did not come to a unanimous conclusion [12,17]. One study performed a separate analysis in SCA patients who underwent multiple surgeries [12]. In this group of SCAs, patients were not found to be at a young age nor did they exhibit other distinct clinical features. 

### 5.3. Recurrence Prediction

One recent retrospective series confirmed that SCAs were 2.9 times more likely to have a tumor residual than ACTH-immunonegative NFPAs [72]. Meanwhile, SCAs have a lower event-free survival [17,72] and a lower progression-free survival [10] compared with other NFPAs. It is worth mentioning that one study observed SCAs have a median recurrence at 3 vs. 8 years for other NFPAs (*p* < 0.0001) [41]. 

Reports have not figured out significant differences in the Ki-67, p53, and matrix metallo-proteinase-9 expression between PAs with and without cavernous sinus invasion [60]. Although, cavernous sinus invasion existed in 85% cases of SCAs in Yamada et al.’s research [60]. However, recent studies have confirmed that the Ki-67 index can be a valuable predictor when surrounding structures are invaded [73]. Additionally, the combination of mitotic count, Ki-67, and p53 was confirmed to be prognostic, especially when combined with invasive tumor growth [74,75,76]. Several cell-cycle regulators, such as p16, cyclin D1, and pRb, were reported to be associated with NFPAs progression [77]. Other reports have suggested that male gender, MIB index ≥ 3%, and SCA tumor pathology serve as significant factors predicting recurrence [17].

### 5.4. Transformation into CD

Several studies have demonstrated that SCAs may transform into CD [11,40], which usually occur in the later course of SCAs [11]. Although the number of cases with transformation is very small, it necessitates attention because it indicates malignant tumor behavior [78]. A retrospective series reported 3.9% of the corticotroph cell adenomas have the ability to bidirectionally transform the phenotype from SCA to CD [39]. Reports have identified that transformation may be more prevalent in Type 1 SCAs than in Type 2 [11]. Zheng et al. retrospectively collected and analyzed 16 patients with SCAs that converted to typical Cushing’s disease [40]. Their research unveiled SCAs that are aggressive and recurrence many times despite receiving multiple treatments tend to transform into CD during follow-up. Results also suggest that all patients that transformed to CD were macroadenomas [40]. Rotman et al. reported the first case of CD transforming into silent corticotroph pituitary carcinoma [79]. Nine SCAs cases who transformed into pituitary carcinoma were reported in a recent systematic review [78]. 

## 6. Management and Prognosis

Kim et al. reported that recurrence rates between SCAs and hormonally negative adenomas (HNAs) were not statistically different when gross total resection (GTR) had been achieved [65]. However, the new WHO classification was not used in this retrospective series, which questions the reliability of the conclusion. The utility of 3T-MRI in endoscopic transsphenoidal surgery may help to achieve GTR of pituitary macroadenomas [80]. However, the aggressive course of SCAs underscores a need for complementary treatments in the postoperative period. Studies have confirmed that radiotherapy, targeted therapies, and cytotoxic chemotherapy, etc. may be useful in the management of SCAs [4,81].

### 6.1. Radiotherapy

Some reports prove that there is no need for prophylactic radiotherapy following GTR in SCAs [10,65]. For those SCAs that did not achieve GTR, early postoperative adjuvant radiosurgery is highly recommend for residual tumor [10]. Prendergast et al. reported a 13-year-old boy diagnosed with SCAs received proton therapy after subtotal gross resection [82]. At the 12-month follow-up post-radiotherapy, MRI showed minimal residual disease, which was stable for 24 months post-radiotherapy [82]. In a study of 104 NFPAs patients treated with stereotactic radiosurgery, 62% of the 34 patients with SCAs achieved tumor control [83]. Progression-free survival (PFS) rates were 73% at 3 years and 31% at 8 years in SCA patients compared with 94% at 3 years and 87% at 8 years in NFPAs patients. Improved PFS was observed at doses >17 Gy [83]. 

### 6.2. Targeted Therapies

In view of the molecular characteristics of SCAs, targeted therapies may be a promising choice [4,81]. The higher expression of membrane SSTR1 and SSTR2 compared to CD by immunohistochemical analysis [58], suggesting somatostatin receptor ligands (SRLs), like PAsireotide, a second-generation SRL, may serve as a promising drug for the treatment of SCAs, particularly in refractory SCAs. However, the efficiency of SRLs for SCAs has not yet been proved. The phase II randomized clinical trial (PASSILCORT; PAsireotide LAR therapy of SCA; NCT02749227) for residual or recurrent SCAs has been terminated and no conclusion has been reached.

### 6.3. Chemotherapy

Several studies have discovered the low expression of O6-methylguanine-DNA methyltransferase (MGMT) in SCAs [66,81,84,85]. Salehi et al. investigated the expression of MGMT in a range of corticotroph adenoma subtypes [84]. In their research, seven SCAs cases showed only 10% MGMT immunohistochemical staining, indicating SCAs, particularly in refractory cases, may be appropriate candidates for temozolomide therapy [84]. Some studies have reported cases of SCAs treated with temozolomide [40,86,87]. In Zheng’s study, four patients with refractory functional SCA received temozolomide [40]. Serum cortisol levels returned to normal in all four cases, and the tumor volume decreased in three of them. Decaroli et al. reported a case of a 78-year-old female who was initially diagnosed with SCA and transformed to CD during the later course [86]. This patient had already received surgery, cabergoline, PAsireotide, and external radiation (a total dose of 54 Gy) before application of temozolomide. However, all these treatments showed an unsatisfactory effect. After being treated with temozolomide for a total of 47 cycles, the tumor volume remarkably decreased, and the serum cortisol levels progressively decreased. Therefore, when therapies like radiotherapy or surgical attempts may be unsuccessful, temozolomide should be considered [87].

## 7. Conclusions

SCAs commonly manifest as NFPAs. Unlike their functioning counterparts, CD, SCAs present no symptoms of hypercortisolism. The diagnosis of SCAs is dependent on a retrospective fashion with histopathological staining of transcription factors and pituitary hormones. SCAs are among the five types of “high-risk” tumors proposed by the 2017 WHO classification system. Studies of the molecular mechanisms related to SCA pathogenesis will provide new directions for the diagnosis and management of SCAs.

## Figures and Tables

**Table 1:** Case series of SCAs.

References	Year	SCA Cases	NFPA Control Group	SCAs Percentage of NFPAs	Classification with TPIT	TPIT+ ACTH+	TPIT+ ACTH-	Hypopituitarism		Recurrence	Progression	Follow-Up Time	Recurrence Predictors
								Preoperative	Postoperative				
1 [14]	2015	83	516	16%	YES	51	32	NA	NA	NA	NA	NA	NA
2 [62]	2018	37	341	9.7%	NO	NA	NA	NA	NA	0/30	3/7	17.95 ± 14.86 M	NA
3 [63]	2010	28	134	17.2%	NO	NA	NA	13	16	7	NA	5.6 ± 4.9 Y	Younger age, male gender, multiple recurrences
4 [12]	2012	33	126	20.8%	NO	NA	NA	25	24	2/16	8/17	42.5 M	NA
5 [72]	2019	41	319	11.3%	NO	NA	NA	NA	NA	2	NA	1.7 Y	NA
6 [64]	2012	20	30	5–6%	NO	NA	NA	NA	NA	Recurrence/re-growth 14%		41 M	NA
7 [71]	2003	28	60	NA	NO	NA	NA	NA	NA	Total recurrence 9/28 (32%)		7.4 Y	NA
8 [17]	2021	62	238	20.6%	YES	57	5	5 Hypocortisolism	6 Hypocortisolism	1/19	19/39	48.4 M	Male gender; MIB index ≥ 3%; SCA tumor pathology
9 [57]	2021	20	137	12.7%	YES	14	6	NA	NA	NA	NA	NA	NA
10 [65]	2019	55	411 HNA	NA	NO	NA	NA	64.7%	25/34	0/35	7/20	49 M	NA
11 [10]	2021	100	841	11.9%	NO	NA	NA	24	NEW 23	0/42	12/58	34.8 M	NA
12 [15]	2020	105	757	24.3%	YES	39	66	38	82	2/46	NA	17.2 ± 7.6 M	NA
13 [9]	2021	112	198 SGAs	30.2%	YES	79	33	20	33	1/74	10/38	14.1 ± 4.6 M	NA

HNA: hormone negative adenomas; SGAs: silent gonadotroph adenomas.
